# Evaluation of Novel Multiplex Antibody Kit for Human Immunodeficiency Virus 1/2 and Hepatitis C Virus Using Sol-Gel Based Microarray

**DOI:** 10.1155/2015/837296

**Published:** 2015-09-17

**Authors:** Seung Gyu Yun, Jin Woo Jang, Jong Han Lee, Chae Seung Lim, Jinhong Kim, Yeona Ki, Minjoung Jo, Soyoun Kim

**Affiliations:** ^1^The Armed Forces Medical School, Daejeon, Republic of Korea; ^2^Department of Laboratory Medicine, Kangwon National University College of Medicine, Chuncheon, Republic of Korea; ^3^Department of Laboratory Medicine, Korea University Medical College, Seoul, Republic of Korea; ^4^PCL, Inc., Seoul, Republic of Korea; ^5^Lab of Nanobiotechnology, Dongguk University, Seoul, Republic of Korea

## Abstract

*Background*. Microarrays enable high-throughput screening (HTS) of disease-related molecules, including important signaling proteins/peptides and small molecules that are in low abundance. In this study, we developed a multiplex blood bank screening platform, referred to as the Hi3-1 assay, for simultaneous detection of human immunodeficiency virus 1/2 (HIV 1/2) and hepatitis C virus (HCV). *Methods*. The Hi3-1 assay was tested using four panels (Panel 1, *n* = 4,581 patient samples; Panel 2, *n* = 15 seroconversion samples; Panel 3, *n* = 4 performance samples; and Panel 4, *n* = 251 purchased positive control samples), and the results were collected by the Department of Laboratory Medicine, Korea University Medical College, Republic of Korea. The present study compares the sensitivity of the multiplex detection platform for both HIV and HCV using a sol-gel based microarray, which was based on a reference test (Architect HIV Ag/Ab Combo and Architect anti-HCV assays), in Korean patients. *Results*. The sensitivity of the multiplex detection platform for both HIV and HCV was 100%, and the specificity was 99.96% for HIV and 99.76% for HCV, which is equivalent to that of the reference test. *Conclusion*. We have successfully applied a novel screening technology to multiplex HIV and HCV diagnoses in a blood bank screening test.

## 1. Introduction

Human immunodeficiency virus (HIV) and hepatitis C virus (HCV) are blood-borne viruses that have proved to be major risk factors for viral transfusion transmitted infection (TTI). HIV and HCV transmission are known to be associated with transfusion of infected blood products, intravenous drug abuse, vertical infection, and sexual contact. HCV causes severe complications after transfusion of contaminated blood. HCV infection is estimated to have a global prevalence of approximately 2%, with 185 million individuals chronically infected with the virus, with 3 to 4 million individuals newly infected each year [1]. Approximately 35 million people are infected with HIV globally. Worldwide, there were approximately 2.1 million new cases of HIV in 2013 [2].

In a recent study, the residual risk of TTI from blood donations in Korea from 2009 to 2010 was estimated to be 1 in 1,356,547 donations for HIV and 1 in 2,984,415 for HCV [3]. All blood donations at the Korean Red Cross Blood Center are screened for HIV and HCV using enzyme immunoassays and nucleic acid amplification tests (NAT).

Conventional enzyme immunoassays (EIA) are the most utilized screening technique, and they have a high sensitivity and are capable of high-throughput sample processing. However, such methods cannot be employed for multiplex antibody tests. Comparatively, protein microarrays can be developed to conduct multiplexing assessments of infectious agents [4].

Over the past decade, microarray technologies have resulted in a paradigm shift in modern biology. Microarrays enable high-throughput screening (HTS) of disease-related molecules, including important signaling proteins/peptides and small molecules that are in low abundance. Such endeavors require excellent molecular binding performance, with high sensitivity, selectivity, and low signal to noise ratio. The sol-gel based microarray technology meets the above-mentioned requirements [5, 6]. Using this technology, we developed a multiplex blood bank screening platform for simultaneous detection of HIV 1/2 and HCV. In this study, we evaluated the performance of the new multiplex platform, which is referred to as the Hi3-1 Multiplex kit (PCL, Inc., Seoul, Republic of Korea), for the detection of HIV and HCV as compared to commercially available chemiluminescent microparticle immunoassays (CMIA).

## 2. Materials and Methods

### 2.1. Materials

The Hi3-1 Multiplex HIV 1/2 and HCV antibody detection kit was tested on four panels and results were collected by the Department of Laboratory Medicine, Korea University Medical College, Republic of Korea.

The first panel (*n* = 4,581) was collected at the Korea University Guro Hospital between May 2009 and October 2010 and consisted of samples collected from hospital patients. All samples were subjected to the following tests: Architect HIV Ag/Ab Combo and anti-HCV immunoassay (Abbott Laboratories, Abbott Park, IL, USA). There were 102 samples that were anti-HIV 1/2 positive, 431 samples that were anti-HCV positive, and 4048 samples that were both anti-HIV and anti-HCV negative based on the Abbott Architect HIV Ag/Ab Combo and the anti-HCV immunoassays. The Abbott Architect HIV Ag/Ab Combo assay for simultaneous qualitative detection of the HIV p24 antigen and antibodies to HIV 1 and/or 2 in human serum and plasma were based a CMIA and used according to the manufacturer's instructions. For detection of HCV, the Abbott Architect anti-HCV assay, which is composed of a recombinant HCV antigen such as Core Ag, NS3, and NS4 and is also based on CMIA, was used according to the manufacturer's instructions. Abbott Architect anti-HCV test qualitatively detected antibodies against HCV.

In order to evaluate the detection capabilities of the Hi3-1 Multiplex HIV 1/2 and HCV antibody detection kit during early infection, the second panel consisted of seroconversion samples. A total of 43 samples from six seroconversion panels (PRB955, PRB958, PRB966, PRB967, PRB968, and PRB972; SeraCare, MA, USA, and Boston Biomedical, MA, USA) were evaluated for HIV infection. A total of 59 samples from nine seroconversion panels (PHV912, PHV913, PHV917, PHV920, PHV921, PHV922, PHV923, PHV924, and PHV925; SeraCare, MA, USA) were tested for HCV infection.

The third panel was a performance panel that was used to evaluate the new Multiplex kit with well-characterized specimens and to provide comprehensive data for HIV and HCV. A total of 36 samples from two performance panels (PRB205 and PRF203; SeraCare, MA, USA) were tested to examine the capability of detection for HIV. A total of 39 samples from two performance panels (PHV106 and PHV207; SeraCare, MA, USA) were tested to examine the capability of detection for HCV.

The fourth panel was obtained from Trina Bioreactives AG, Nänikon, Switzerland, in order to evaluate the performance of the new Multiplex kit according to HIV subtypes and HIV-Ab. Positive human plasma samples were obtained from blood donors. From this panel, 150 samples were HIV type 1 and 100 samples were HIV type 2 and 1 HIV 1 type.

### 2.2. Multiplex Antibody Kit Using Sol-Gel Based Microarray

**Figure 1:**
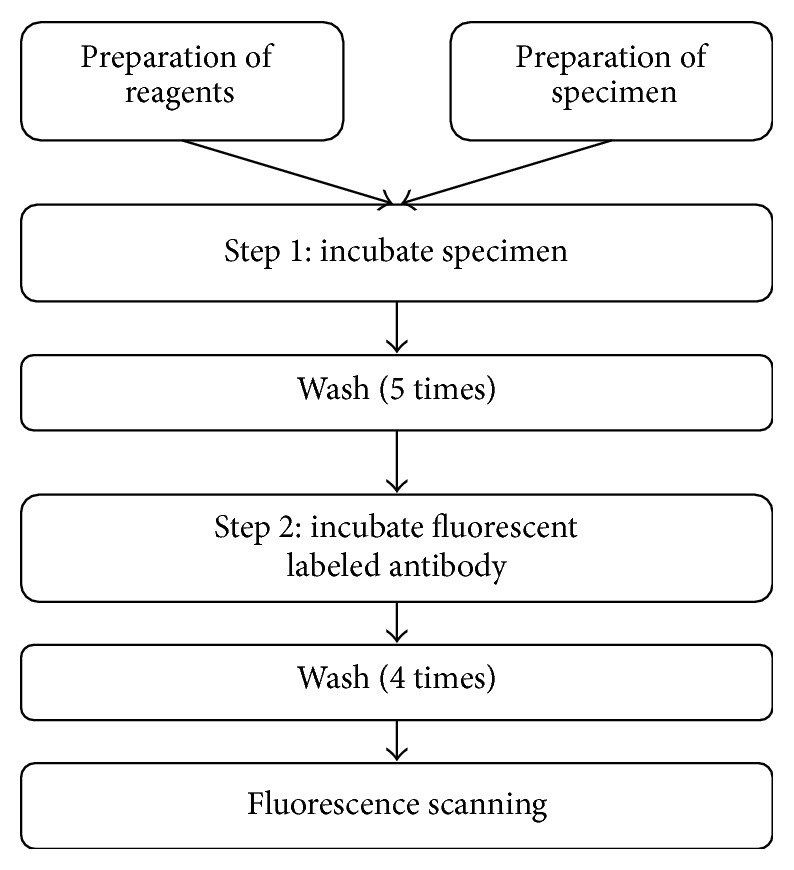
Schematic diagram of examination procedure on the Hi3-1 Multiplex test.

**Figure 2:**
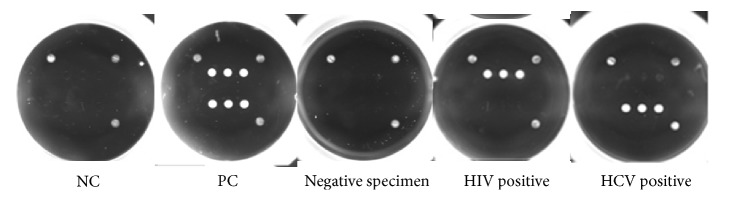
Representative scanned images of the Hi3-1 Multiplex kit. For the manufacturing protein microarray, 5 different HIV 1/2 and O type antigens were mixed with a sol-gel solution spotted onto a well of 96-well plate (upper spots). Four different HCV antigens were also mixed with a sol-gel solution and spotted onto the same well as the HIV antigens spots (lower spots). ^*∗*^NC: negative control; PC: positive control.

**Figure 3:**
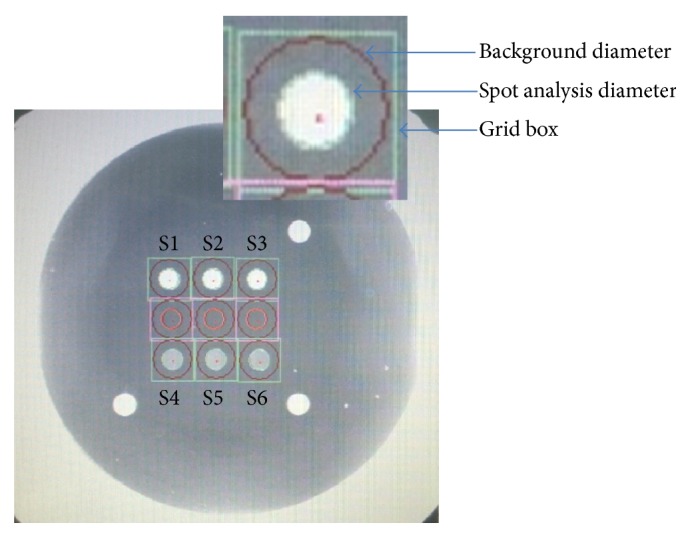
Specifying the size and position of the spots in the scanned image. There are 9 spots, including triplets for HIV 1/2 (S1, S2, and S3), HCV (S4, S5, and S6), and 3 positioning markers. Measurement of the signal intensity of the specified spots (arrow, spot analysis diameter) and background (arrow, background diameter) are shown. Upper spots (S1–S3) consisted of an HIV recombinant antigen mixture containing p24, gp36, gp41 (M type, O type), and gp120. Lower spots (S4-S5) consisted of a HCV recombinant antigen mixture containing Core, NS3, NS4, and NS5.

The Hi3-1 assay is a two-step fluorescence-based immunoassay used to determine the presence of antibodies of HIV 1/2 and/or HCV in human serum or plasma (Figure 1). On the bottom of the microwells of the Hi3-1 kit, sol-gel spots are arrayed, and antigens for HIV 1/2/O and HCV are encapsulated within each spot. Human serum or plasma that has been diluted with specimen diluent is added to the wells along with negative and positive controls. HIV and HCV antibodies in serum or plasma bind to antigens in the sol-gel spots (Step 1). Following a wash cycle, fluorescence-labeled secondary antibodies against human IgG and IgM are added to the wells. During this incubation step, antibodies present in the sample are bound to the sol-gel spots in an antigen-antibody-secondary antibody-fluorescence complex (Step 2). In the absence of HIV and HCV antibodies, no fluorescence is detected. After washing to remove samples and unbound fluorescently labeled antibodies, the microwell plate is scanned with a fluorescence microplate scanner (Figure 2). The Hi3-1 assay assessment times and conditions used for the automated ELISA equipment were similar to conventional ELISA diagnostic assays. There are nine spots—triplets for HIV 1/2 (S1, S2, and S3), HCV (S4, S5, and S6), and three positioning markers (Figure 3). The signal intensity of specified spots and background are measured, S-BG is calculated by subtraction of the background signal intensity from the spot signal intensity, and the average of S1, S2, and S3 for HIV 1/2 and S4, S5, and S6 for HCV was quantified. The signal intensity was quantified as the “median” intensity of the spot. Analysis software was used to calculate the value of S-BG, NCx, NCy, and S/Co using the following formulations: S-BG = (average signal intensity of S1~S3 or S4~S6) − (Average background signal intensity), NCx = Average of (S-BG) of HIV 1/2 (S1~S3) from the Negative control (wells A1, B1), NCy = average of (S-BG) of HCV (S4~S6) from the Negative control (wells A1, B1), S/CoHIV = (S-BGHIV)/(NCx + 0.3), S/CoHCV = (S-BGHCV)/(NCy + 0.3). The cut-off value for S/Co (signal to cut-off value) was determined using empirical analysis of negative samples. According to the test results, the mean, 3 standard deviations (SD), and the highest signal of the negative samples were 0.0826, 0.1094 and 0.3613 for HCV and 0.0826, 0.092, and 0.3157 for HIV, respectively. The cut-off values were set between the mean plus 3 SD and the maximum value of the negative samples in the collection, and the value was chosen to be 0.3 considering both specificity and sensitivity. Therefore, we established the cut-off value as the median intensity of negative control plus 0.3. For interpretation of the results, if S/CoHIV or S/CoHCV was greater than or equal to 1, we concluded that HIV 1/2 or HCV antibody was detected.

### 2.3. Reference Methods

The Multiplex antibody kits used as reference assays were the Architect HIV Ag/Ab Combo and Architect anti-HCV assays. The samples that were nonreactive for both the Multiplex antibody kit and the Architect kit were classified as negative, and no confirmatory tests were performed on these samples. In addition, the samples that were reactive to both the Multiplex antibody kit and the CMIA were classified as positive, without confirmatory tests. Discrepant samples were verified using the HIV western blot kit or the HCV recombinant immunoblot assay (RIBA). The samples with negative western blot or RIBA test results were considered negative, and the samples with indeterminate results with these confirmatory methods were excluded from this analysis.

## 3. Results

Concordance of the corresponding HIV Ab and HCV-Ab assays between the Hi3-1 and Architect systems for panel 1 was 99.96% and 99.78%, respectively. Despite the remarkable correlation, some discrepant results were observed (Table 1). Two out of 4,581 samples were discordant for HIV Ab, and 10 out of 4,581 were discordant for HCV-Ab. All discordant samples detected using the Hi3-1 system were identified as false positives using confirmatory HIV western blot analysis or HCV RIBA. Overall, for the Hi3-1 system the sensitivity of both the HIV-Ab and HCV-Ab assays was 100.00%, and the specificities for the HIV-Ab and HCV-Ab assays were 99.96% and 99.76%, respectively.

The ability of the Hi3-1 Multiplex kit to detect anti-HIV and anti-HCV was evaluated by testing six HIV and nine HCV seroconversion panels from blood and plasmapheresis donors who seroconverted over the course of their donation history. The Hi3-1 Multiplex kit detected anti-HIV 8 days later than the Architect HIV Ag/Ab Combo assay for one of the panels and 4 days later for another of the six panels. The Hi3-1 multiplex kit detected anti-HCV 3 days earlier than the Architect anti-HCV for one of the panels and 7 days or 9 days earlier for three of the other nine panels. In particular, the Hi3-1 multiplex kit detected anti-HCV earlier than any other anti-HCV screening test, according to supplier data, for the PHV913, PHV920, and PHV921 panels. Both the Hi3-1 multiplex and Architect assays exhibited equivalent detection of anti-HIV and anti-HCV for 9 of the 15 panels (Table 2).

The sensitivity of the Hi3-1 Multiplex kit was assessed using four different commercially available performance panels (Table 3). One panel member of the anti-HIV 1 mixed titer panel (PRB205-24) was determined to be nonreactive using the Hi3-1 multiplex kit. Supplier data stated that this panel member was reactive using the Abbott Anti-HCV HIV-1/HIV-2 rDNA, 2.0 and Ortho HIV-1/HIV-2 Vitros ECi, and Siemens HIV 1/0/2 Enhanced Advia Centaur assays; however, the panel was negative using the HIV Bio-Rad Western Blot, Avioq HIV-1 Microelisa System, and Bio-Rad HIV-1/HIV-2 Plus O Gen Sys assays.  Table 4 shows detailed data corresponding to samples with discordant results between the Hi3-1 Multiplex and the Abbott anti-HCV assays. In the case of the HCV panel, five members of the anti-HCV low titer panel (PHV106-1, 106-3, 106-13, 106-14, and 106-15) and one member of the anti-HCV mixed titer panel (PHV207-2) presented discordant results. Among the discordant results, three members (PHV106-1, 106-13, and 106-14) of the anti-HCV low titer panel and one member of the anti-HCV mixed titer panel changed from reactive using the Abbott anti-HCV assay to nonreactive using the Hi3-1 Multiplex kit. However, two members (PHV106-3 and 106-15) changed from reactive with the Hi3-1 Multiplex kit into nonreactive with the Abbott anti-HCV assay. All sample switching from nonreactive with the Hi3-1 multiplex kit to reactive with the Abbott anti-HCV assay exhibited isolated reactivity for either anti-NS3 or anti-NS4 as assessed using an immunoblot test. Two samples that switched to reactive with the Hi3-1 Multiplex kit displayed reactivity for anti-Core, as detected using an immunoblot test.

In addition, the Hi3-1 Multiplex kit is capable of detecting HIV/HCV coinfection, as assessed using samples from panel 3. For example, various panel samples from PRB205 were positive for anti-HCV (members 2 and 16) and were correctly tested as reactive for both HIV and HCV using the Hi3-1 Multiplex kit. Likewise, samples from PHV106 were positive for anti-HIV (members 9 and 12) and were correctly tested as reactive for both HIV and HCV using the Hi3-1 Multiplex kit.

The new multiplex platform exhibited 100% sensitivity for detection of HIV 1, HIV 2, and HIV 1 O subtypes, while cross reactivity was not observed (Table 5). Thus, the Hi3-1 Multiplex system was able to detect all subtypes in a similar fashion to the existing Architect assay. The new platform is compatible with the current CLIA based automated system, which is used for blood bank screening.

## 4. Discussion

As a result of improved screening reagents, implementation of the nucleic acid amplification test, and tight application of strict donor selection procedures, the risk of viral TTI has decreased remarkably over the past 10 years [3]. However, there still remains a residual risk of viral TTI. To prevent TTI, blood centers have continuously performed screening tests for blood donors worldwide. At present, the American and Korean Red Cross Blood Centers are using several commercially available EIA and CMIA kits to screen for HIV and HCV [3]. Among several assays for the detection of viral TTI that are commercially available, the Architect system has been frequently studied in published articles [7–11]. Therefore, we chose the Architect system to compare to the Hi3-1 Multiplex kit. EIA and CMIA assays that have been used commonly in blood centers have a disadvantage in that they have not been developed into multiplexing methods. To address this, we have developed a sol-gel based protein microarray that can perform multiplexing to screen for both HIV and HCV in blood banks.

The results indicate that the newly developed Hi3-1 Multiplex kit, which is based on a sol-gel protein microarray, achieves a similar capability of detection for viral Ab when compared to the commercial Architect assays. In the present study, the concordance of the corresponding HIV Ab and HCV Ab assays between the Hi3-1 system and the Architect systems for panel 1 was 99.96% and 99.78%, respectively. Out of 4,581 samples (panel 1), 12 (2 HIV Ab and 10 HCV Ab) samples showed different results in the two screening assays. The use of confirmatory tests, including HIV western blot analysis or HCV RIBA, resolved this issue with negative immunoblot test results.

These samples had low S/Co values (range of 1.19 to 3.54) and tended to yield false-positive results using the Hi3-1 assay (Table 1), whereas with the Architect assays, these samples were shown to be negative for HIV and HCV. Most studies compare the specificity of anti-HIV and anti-HCV assays by screening healthy individuals such as blood donors [7]. However, patient samples showed lower specificity than the samples of blood donors. Due to various clinical conditions, including autoimmune diseases, nephritic syndrome, liver diseases, blood transfusion, hemodialysis, multiple pregnancies, and other viral infections, anti-HIV and/or anti-HCV screening assays may generate false-reactive results [7, 12–14]. In this study, the Hi3-1 assay lacked specificity for various samples; unfortunately, we did not perform additional tests to establish the cause of the false reactions and did not collect clinical characteristics of subjects with false-positive results for the Hi3-1 assay. According to our results, this novel multiplexing kit requires improvement for false-positive reactions, but overall, the sensitivity for the commercially available anti-HIV and anti-HCV seroconversion panel (panel 2) was quite similar to that of the Architect assays. Seroconversion sensitivity for the Hi3-1 assay was slightly different compared to the conventional Architect assay when six HIV and nine HCV seroconversion panels were examined. For two of the six HIV panels, the Architect assay showed higher sensitivity than the Hi3-1 assay. According to a previous study that surveyed early detection capabilities using an HIV seroconversion panel, fourth-generation HIV assays, which detect both HIV antibodies and p24 antigen simultaneously, have shortened the seroconversion window as compared with the third-generation assays, which detect only HIV antibodies [15, 16]. One possible explanation for the differences seen in the sensitivity between the Hi3-1 assay and the Architect HIV Ag/Ab combo assay for the seroconversion panel might be the antigens and/or antibodies included in the assays. However, the Hi3-1 assay demonstrated more rapid detection capabilities than the Architect assay for four of the nine HCV panels. We expect that the inclusion of additional antigens such as NS5 would improve the sensitivity of the Hi3-1 assay. In an earlier study, we determined that a previous microarray version of the Hi3-1 assay had a detection limit that was 1000 times more sensitive than a commercial anti-HCV ELISA [17]. This superior sensitivity of the Hi3-1 assay for the detection of anti-HCV is also supported by the current study.

Analysis of the performance panel revealed that the Hi3-1 multiplex kit was similar in sensitivity to the Architect system. However, in the case of the HCV performance panel, the Hi3-1 assay exhibited reduced detection of a limited number of lower titer samples compared with the Architect anti-HCV assay, which was in contrast to results for the HCV seroconversion panel.  Table 4 includes information about possible explanations for these observations. The Hi3-1 assay can more readily detect anti-Core compared to the Architect anti-HCV assay for two samples. In comparison with the Hi3-1 assay, the Architect anti-HCV assay has a marked improvement for sensitivity, especially in regard to either residual anti-NS3 or anti-NS4 activity for four samples. Since the two kits use HCV antigens from different genomic region sources, this study observed reactivity differences in the low titer panel. Similarly, samples in the HCV seroconversion panel, which showed early detection as tested using the Hi3-1 assay, suggested the result of isolated reactivity for anti-Core by immunoblot test. Additionally, the Hi3-1 multiplex assay showed 100% sensitivity for the detection of all the HIV subtypes (HIV 1, HIV 2, and HIV 1 O), while cross reactivity was not observed. These results indicate that the accuracy of the Hi3-1 assay is nearly equivalent to that of the CMIA-based Architect assays.

Previously, several groups have reported on the use of rapid diagnostic tests with multiple detection capabilities such as HIV/HCV/TB and HIV/HCV/ABO blood type assays [18–20]. However, these assays were developed to optimize particularly the value in a resource limited setting, rather than for use in large-scale screening. To our knowledge, the Hi3-1 assay is the first multiplexing test that can be utilized in an automated and high-throughput system due to its cost-effective and time-saving aspects. High-throughput methods do exist that can measure HIV or HCV separately, such as the Abbott Architect system. However, there is no existing multiplex method for simultaneous HIV and HCV detection that is currently available. Thus, compared with existing high-throughput methods, we can reduce the time and cost by half as the result of the multiplexity of the two parameters (HIV and HCV).

However, there is one important limitation. Most of the samples analyzed in our study were obtained from patients individually infected by HIV or HCV. Researchers have reported that some HCV assays showed an increased amount of false-negative results with HIV infected subjects [21, 22]. Further studies are required to establish if this is the case for HIV/HCV coinfected samples for diagnostic purposes. However, for blood product screening, it is less crucial to be able to detect HIV/HCV coinfection as any donations screening positive for either HIV or HCV will be discarded [23].

In conclusion, a novel screening technology was successfully utilized as a multiplex HIV and HCV diagnostic tool for blood bank screening. Given that the new technology had sensitivity and specificity equivalent to the commercially available CLIA tests, the sol-gel based microarray has the potential to be used as a high-throughput screening tool for simultaneous detection of HIV and HCV in blood banks.

## Figures and Tables

**Table 1:** Performance of the Hi3-1 Multiplex kit compared against the Architect system on samples of panel 1.

	Abbott diagnostics (i) HIV 1/2: Architect HIV Ag/Ab Combo test (ii) HCV: Architect anti-HCV		Concordance rate (%)
	Positive^*∗*^	Negative	
Hi3-1 Multiplex HIV 1/2			
Positive	102	2	99.96
Negative	0	4477	
Hi3-1 Multiplex HCV			
Positive	431	10	99.78
Negative	0	4140	

^*∗*^HIV or HCV only-positive specimens were included.

**(a):** 

HIV panel	Bleed day of first positive result by the Hi3-1 Multiplex kit	Bleed day of first positive result by the Architect HIV Ag/Ab Combo
PRB955	12	12
PRB958	**15**	**7**
PRB966	**48**	**44**
PRB967	17	17
PRB968	26	26
PRB972	18	18
Total number of bleeds	43	43
Detected bleeds	15	18

**(b):** 

HCV panel	Bleed day of first positive result by the Hi3-1 Multiplex kit	Bleed day of first positive result by the Architect anti-HCV
PHV912	**4**	**7**
PHV913	**0**	**7**
PHV917	85	85
PHV920	**7**	**16**
PHV921	**0**	**7**
PHV922	3	3
PHV923	21	21
PHV924	59	59
PHV925	27	27
Total number of bleeds	59	59
Detected bleeds	42	35

The number of detected bleeds was calculated. Discrepant results between the Hi3-1 and Architect system are highlighted in bold.

**Table 3:** Comparison of the sensitivity between the Hi3-1 Multiplex and the Architect system with performance panel sera (panel 3).

Performance panel	Number of panel members	The Hi3-1 Multiplex kit	The Architect system
PRB205 (anti-HIV 1 mixed titer panel)	25	22	23
PRF203 (anti-HIV 2 panel)	11	10	10
PHV106 (anti-HCV low titer panel)	14	10	11
PHV207 (anti-HCV mixed titer panel)	25	22	23

Two samples of PRB205, one sample of PRF203, one sample of PHV 106, and two samples of PHV207 are characterized by the supplier as negative and were tested nonreactive by the Hi3-1 and the Architect assay.

**Table 4:** Discordant results obtained by the Hi3-1 Multiplex and the Architect anti-HCV assay from HCV performance panel.

Sample number	The Hi3-1 Multiplex kit (S/CO)	The Architect anti-HCV (S/CO)	CHIRON RIBA HCV 3.0 SIA pattern	INNO-LIA HCV Score pattern	Ortho RIBA 3.0 pattern
PHV106-01	0.24	2.6	NS3 1+	NS3 2+	—
PHV106-13	0.50	1.3	NS4 3+	Not detected	—
PHV106-14	0.54	2.0	NS4 3+	Not detected	—
PHV207-02	0.35	3.3	—	—	NS3 1+
PHV106-03	8.48	0.6	Core 1+	Core1 1+	—
PHV106-15	8.58	0.4	Core 2+	Core1 2+	—

^*∗*^—: not tested on the sample.

**Table 5:** Performance evaluation for HIV subtypes samples (panel 4) on the Hi3-1 Multiplex kit.

			Purchased sample from Trina (i) HIV 1: INNO-LIA HIV I/II Score Innogenetics (ii) HIV 2: Inverness Medical HIV I/II Ag/Ab Combo (iii) HIV 1 O type: in-house sequencing		Concordance rate (%)
			Positive	Negative	
Hi3-1 Multiplex test	HIV 1	Positive	150	0	100.00
		Negative	0	0	
	HIV 2	Positive	100	0	100.00
		Negative	0	0	
	HIV 1 O type	Positive	1	0	100.00
		Negative	0	0	

## Abbreviations

HCV: Hepatitis C virus
HIV: Human immunodeficiency virus
HTS: High-throughput screening
TTI: Transfusion transmitted infection
CMIA: Chemiluminescent microparticle immunoassays
NAT: Nucleic acid amplification test
EIA: Enzyme immunoassays
RIBA: Recombinant immunoblot assays.

## References

[1] WHO (2014). *Guidelines for the Screening, Care and Treatment of Persons with Hepatitis C Infection*.

[2] WHO HIV/AIDS Fact sheet no. 360. http://www.who.int/mediacentre/factsheets/fs360/en/.

[3] Kim M. J., Park Q., Min H. K., Kim H. O. (2012). Residual risk of transfusion-transmitted infection with human immunodeficiency virus, hepatitis C virus, and hepatitis B virus in Korea from 2000 through 2010. *BMC Infectious Diseases*.

[4] Petrik J. (2010). Microarray blood testing: pros & cons. *Biologicals*.

[5] Kim S., Kim Y., Kim P. (2006). Improved sensitivity and physical properties of sol-gel protein chips using large-scale material screening and selection. *Analytical Chemistry*.

[6] Lee S., Kim Y. S., Jo M., Jin M., Lee D.-K., Kim S. (2007). Chip-based detection of hepatitis C virus using RNA aptamers that specifically bind to HCV core antigen. *Biochemical and Biophysical Research Communications*.

[7] Berger A., Rabenau H., Allwinn R., Doerr H. W. (2008). Evaluation of the new ARCHITECT anti-HCV screening test under routine laboratory conditions. *Journal of Clinical Virology*.

[8] Echevarría J. M., Avellón A., Jonas G., Hausmann M., Vockel A., Kapprell H.-P. (2006). Sensitivity of a modified version of the ARCHITECT anti-HCV test in detecting samples with immunoblot-confirmed, low-level antibody to hepatitis C virus. *Journal of Clinical Virology*.

[9] Jonas G., Pelzer C., Beckert C., Hausmann M., Kapprell H.-P. (2005). Performance characteristics of the ARCHITECT Anti-HCV assay. *Journal of Clinical Virology*.

[10] Krawczyk A., Hintze C., Ackermann J. (2014). Clinical performance of the novel DiaSorin LIAISON XL murex: HBsAg quant, HCV-Ab, HIV-Ab/Ag assays. *Journal of Clinical Virology*.

[11] Chavez P., Wesolowski L., Patel P., Delaney K., Owen S. M. (2011). Evaluation of the performance of the Abbott ARCHITECT HIV Ag/Ab Combo Assay. *Journal of Clinical Virology*.

[12] Mahajan V. S., Pace C. A., Jarolim P. (2010). Interpretation of HIV serologic testing results. *Clinical Chemistry*.

[13] Erickson C. P., McNiff T., Klausner J. D. (2006). Influenza vaccination and false positive HIV results. *The New England Journal of Medicine*.

[14] Chao T. T., Sheffield J. S., Wendel G. D., Ansari M. Q., McIntire D. D., Roberts S. W. (2012). Risk factors associated with false positive HIV test results in a low-risk urban obstetric population. *Journal of Pregnancy*.

[15] Cohen M. S., Gay C. L., Busch M. P., Hecht F. M. (2010). The detection of acute HIV infection. *The Journal of infectious diseases*.

[16] Lee K., Park H.-D., Kang E.-S. (2013). Reduction of the HIV seroconversion window period and false positive rate by using ADVIA Centaur HIV antigen/antibody combo assay. *Annals of Laboratory Medicine*.

[17] Kwon J.-A., Lee H., Lee K. N. (2008). High diagnostic accuracy of antigen microarray for sensitive detection of hepatitis C virus infection. *Clinical Chemistry*.

[18] Lochhead M. J., Todorof K., Delaney M. (2011). Rapid multiplexed immunoassay for simultaneous serodiagnosis of HIV-1 and coinfections. *Journal of Clinical Microbiology*.

[19] Corstjens P. L. A. M., Chen Z., Zuiderwijk M. (2007). Rapid assay format for multiplex detection of humoral immune responses to infectious disease pathogens (HIV, HCV, and TB). *Annals of the New York Academy of Sciences*.

[20] Burgess S. T. G., Kenyon F., O'Looney N. (2008). A multiplexed protein microarray for the simultaneous serodiagnosis of human immunodeficiency virus/hepatitis C virus infection and typing of whole blood. *Analytical Biochemistry*.

[21] Marcellin P., Martinot-Peignoux M., Elias A. (1994). Hepatitis C Virus (HCV) viremia in human immunodeficiency virus-seronegative and -seropositive patients with indeterminate HCV recombinant immunoblot assay. *Journal of Infectious Diseases*.

[22] Bonacini M., Lin H. J., Hollinger F. B. (2001). Effect of coexisting HIV-1 infection on the diagnosis and evaluation of hepatitis C virus. *Journal of Acquired Immune Deficiency Syndromes*.

[23] Kosack C. S., Nick S., Shanks L. (2014). Diagnostic accuracy evaluation of the ImmunoFlow HCV rapid immunochromatographic test for the detection of hepatitis C antibodies. *Journal of Virological Methods*.

